# The complete chloroplast genome of *Epimedium qingchengshanense* G. Y. Zhong et B. L. Guo (Berberidaceae), an endangered species endemic to China

**DOI:** 10.1080/23802359.2020.1715295

**Published:** 2020-01-24

**Authors:** Yanjiao Luo, Xiang Liu, Yao Yu, Qianru Yang, Baolin Guo, Chaoqun Xu, Fengmei Suo, Cheng Zhang, Guoan Shen, Anjia Chen

**Affiliations:** a College of Pharmacy, Shanxi Medical University, Taiyuan, China;; b Institute of Medicinal Plant Development, Chinese Academy of Medical Science, Peking Union Medical College, Beijing, China;; c Chongqing Academy of Chinese Materia Medica, Chongqing, China

**Keywords:** Chloroplast genome, *Epimedium qingchengshanense*, Berberidaceae

## Abstract

*Epimedium qingchengshanense* G. Y. Zhong & B. L. Guo is an endangered species with high ornamental value and medicinal value in China. In this study, we reported the first complete chloroplast (cp) genome of *E. qingchengshanense*. The whole cp genome of *E. qingchengshanense* is 159,087 bp in length, comprising a pair of inverted repeat regions (IRs) (27,709 bp) that are separated by a large single-copy (LSC) region (86,607 bp) and a small single-copy (SSC) region (17,062 bp). The circular genome contains 112 unique genes, of which 78 are protein-coding genes, 30 tRNA, and 4 rRNA genes. Phylogenetic analysis shows that *E. qingchengshanense* has a closer relationship with other *Epimedium* species.

The plant species of the genus *Epimedium* L. are perennial herbs of Berberidaceae. Many species in *Epimedium* family are well-known traditional Chinese medicinal herbs because of their special therapeutic effects on human beings in nourishing kidney-yang, strengthening muscles and bones, and dispelling rheumatism (Jiang et al. 2015; Zhang et al. 2016). In recent years, the research on *Epimedium* family has attracted more attention. *Epimedium qingchengshanense*, an endangered species with many good traits for agricultural cultivation, is narrowly distributed to the Qingcheng Mountain in Dujiangyan City, Sichuan Province (Guo et al. 2007). In this study, we reported the complete chloroplast (cp) genome of this economically important wild species, which provides valuable genetic information for the study of evolutionary dynamics and conservation.

The sample for total genomic DNA extraction was obtained from the fresh leaves of *E. qingchengshanense* that were collected in Dujiangyan of Sichuan Province, China (N30°55′, E103°28′). The voucher samples (16025) were deposited at the Herbarium of the Institute of Medicinal Plant (IMPLAD), Beijing, China. Genomic DNA was extracted using the modified CTAB method (Doyle and Doyle 1987). DNA library preparations were sequenced, and 150 bp paired-end reads were generated on an Illumina Novaseq PE150 platform. The clean reads were assembled using the program GetOrganelle v1.5 (Jin et al. 2018) with the reference chloroplast genome of *E. acuminatum* (GenBank accession number: KU522469), the chloroplast genome annotation was performed through the online program CPGAVAS2 (Shi et al. 2019) and GeSeq (Tillich et al. 2017), followed by manual correction. The annotated genomic sequence has been registered in GenBank with an accession number (MN815805).

The complete chloroplast genome of *E. qingchengshanense* is 159,087 bp in length and exhibits a typical quadripartite structure, and contains two inverted repeat regions (IRa and IRb) of 27,709 bp that is separated by a large single-copy (LSC, 86,607 bp) and a small single-copy (SSC, 17,062 bp). The total GC content of the complete chloroplast genome, LSC, SSC, IR regions is 38.81%, 37.29%, 32.77%, and 43.05%, respectively. The complete chloroplast genome of *E. qingchengshanense* contains 112 unique genes, including78 protein-coding genes, 30 tRNA, 4 rRNA genes and 4 pseudogenes (*ψinfA, ψycf1, ψycf15, and rpl22*). Most of the genes are single-copy genes. However, six protein-coding genes (*rps7, ndhB, ycf2, rp123, rpl2,* and *rps19*), seven tRNAs (*trnI-CAU, trnL-CAA, trnV-GAC, trnI-GAU, trnA-UGC, trnR-ACG*, and *trnN-GUU*), and four rRNAs (*rrn16, rrn23, rrn4.5, and rrn5*) are duplicated in the IR regions. One tRNA gene (*rnQ-UUG*) duplicated in the LSC regions. In these genes, 15 genes (six tRNA genes and nine protein-coding genes) contain one intron, and three genes (*ycf3*, *clpP*, and *rps12*) contain a couple of introns. The *rps12* gene is trans-spliced, with the 5′ end located in the LSC region, and the 3′ end duplicated in the IR region.

To confirm the phylogenetic position of *E. qingchengshanense*, the complete chloroplast genomes of 22 other plant species with *Akebia quinata* as outgroup species were downloaded from the NCBI GenBank database. The sequences were aligned using MAFFT v7 (Katoh et al. 2017), and then the maximum likelihood tree (Figure 1) was constructed using raxmlGUI1.5b (v8.2.10) (Silvestro and Michalak 2012). Phylogenetic analysis shows that *E. qingchengshanense* is closely related to *E. acuminatum* and *E. davidii*. The published *E. qingchengshanense* chloroplast genome provides useful information for phylogenetic and evolutionary studies in Berberidaceae.

**Figure 1.**
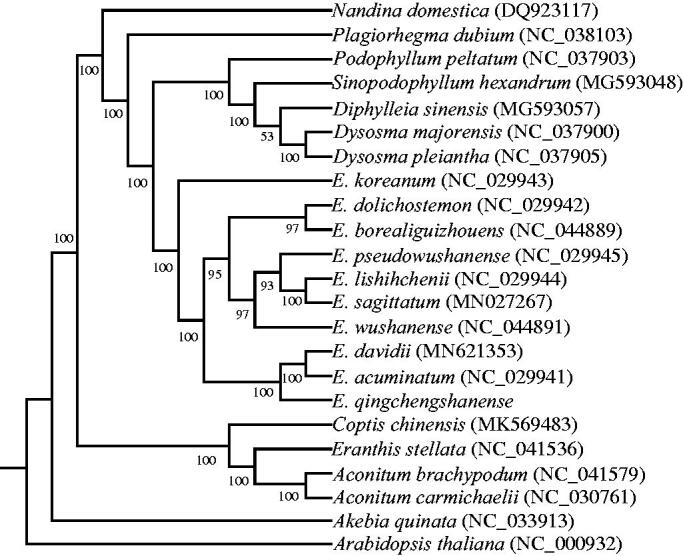
The Maximum likelihood (ML) phylogenetic tree based on complete chloroplast genomes of 23 species, with *Akebia quinata* as outgroup. The numbers above the lines represent ML bootstrap values.
